# The caspase-1 inhibitor AC-YVAD-CMK attenuates acute gastric injury in mice: involvement of silencing NLRP3 inflammasome activities

**DOI:** 10.1038/srep24166

**Published:** 2016-04-07

**Authors:** Fang Zhang, Liang Wang, Jun-jie Wang, Peng-fei Luo, Xing-tong Wang, Zhao-fan Xia

**Affiliations:** 1 Department of Burn Surgery, the Second Military Medical University Affiliated Changhai Hospital, Shanghai 200433, China; 2 Number 73901 Troop of PLA, Shanghai 200439.

## Abstract

This study evaluated the protective effects of inhibiting caspase-1 activity or gastric acid secretion on acute gastric injury in mice. AC-YVAD-CMK, omeprazole, or vehicle were administered to mice before cold-restraint stress- or ethanol-induced gastric injury. Survival rates and histological evidence of gastric injury of mice pretreated with AC-YVAD-CMK or omeprazole, and exposed to cold-restraint stress, improved significantly relative to the vehicle group. The increased levels of tumour necrosis factor-α, interleukin (IL)-1β, IL-6, and IL-18 following cold-stress injury were decreased by AC-YVAD-CMK, but not omeprazole, pretreatment. The increased expression of CD68 in gastric tissues was inhibited significantly by AC-YVAD-CMK pretreatment. Inhibiting caspase-1 activity in the NLRP3 inflammasome decreased gastric cell apoptosis, and the expression of Bax and cleaved caspase-3. AC-YVAD-CMK pretreatment significantly inhibited cold-restraint stress-induced increases in the expression of phosphorylated IκB-alpha and P38. General anatomy and histological results showed the protective effect of AC-YVAD-CMK on ethanol-induced acute gastric injury. Overall, our results showed that the caspase-1 inhibitor AC-YVAD-CMK protected against acute gastric injury in mice by affecting the NLRP3 inflammasome and attenuating inflammatory processes and apoptosis. This was similar to the mechanism associated with NF-κB and P38 mitogen-activated protein kinase signalling pathways.

Acute gastric ulcer is usually a superficial and diffuse mucosal lesion of the stomach. This type of ulcer occurs frequently as a result of alcohol consumption, ingestion of nonsteroidal anti-inflammatory drugs, and major stressful events such as severe burns, surgery, shock, and trauma^1^,^2^. Acute gastric ulcer may result in severe upper gastrointestinal bleeding with high mortality and morbidity^3^. The pathogenesis of acute gastric ulcer is still obscure, but the acute phase of inflammation is considered one of the major etiological factors maintaining and regulating the severity of this disorder^4^,^5^.

Inflammasomes are multi-protein oligomers of the innate immune system. Inflammasomes play an important role in the pathogenesis of various diseases including *Helicobacter pylori* gastritis, gout, diabetes mellitus, and dengue haemorrhagic fever^6^,^7^,^8^. The nucleotide-binding oligomerization domain, leucine rich repeat and pyrin domain-containing (NLRP)3 inflammasome have been studied extensively. The NLRP3 inflammasome contains an adaptor protein, apoptosis-associated speck-like protein containing a caspase recruitment domain (ASC), and an effector protein, caspase-1^9^,^10^. The NLRP3 inflammasome recognizes certain microbial and danger components and serves as a platform for activating caspase-1 and maturation of the pro-inflammatory cytokine, IL-1β. Dinarello *et al*. indicated that proper regulation of the inflammasome could affect the balance between the production of anti- and pro-inflammatory cytokines^11^. Meanwhile, Rozza *et al*. considered that cytokines such as tumour necrosis factor (TNF)-α, interleukin (IL)-6, and IL-10 played important roles in the regulation of acute gastric ulcers^12^,^13^.

Caspase-1 is a key enzyme of the NLRP3 inflammasome, which is a critical pro-inflammatory mediator that modulates host responses to various stress conditions^14^. Therefore, we used the caspase-1 inhibitor, AC-YVAD-CMK, and investigated its protective effects in cold-restraint stress- and ethanol-induced mouse acute gastric injury models. We found that pretreatment with AC-YVAD-CMK attenuated mouse acute gastric injury. This protection involved silencing NLRP3 inflammasome activities and alleviating inflammatory responses and apoptosis. The underlying mechanism was associated with the NF-κB and P38 mitogen-activated protein kinase (MAPK) signalling pathways.

## Results

### AC-YVAD-CMK inhibited caspase-1 activation in the NLRP3 inflammasome

**Figure 1:**
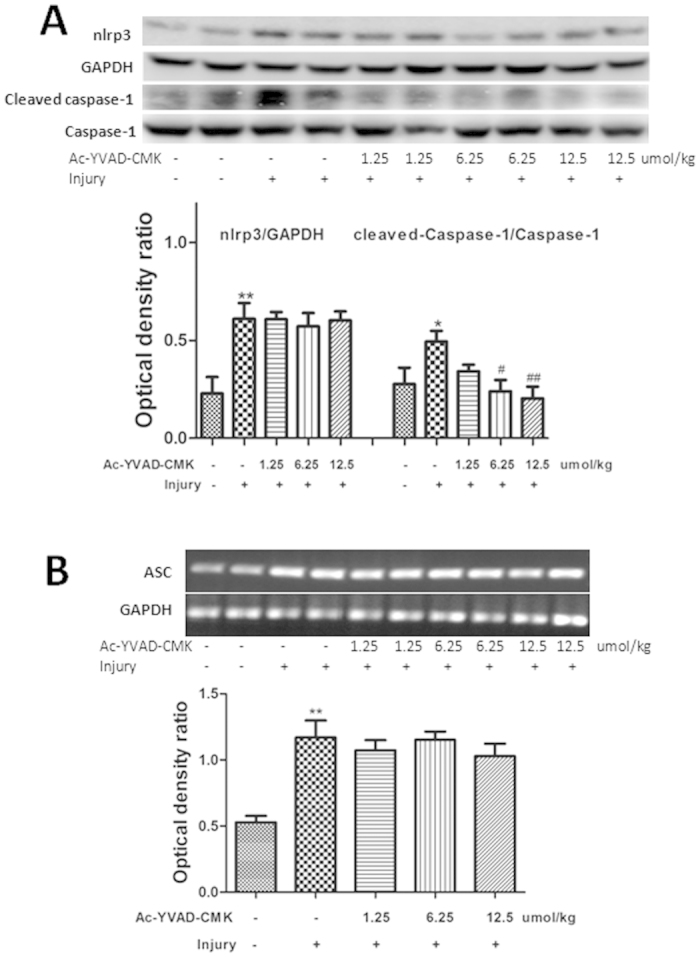
The effects of AC-YVAD-CMK pretreatment on the NLRP3 inflammasome in mice. (**A**) The protein levels of NLRP3 and cleaved caspase-1 were determined by western blotting. Protein levels were quantified by densitometry and are expressed as the optical density ratio to GAPDH. (**B**) mRNA levels of ASC were determined by RT-PCR, quantified by densitometry, and expressed as the optical density ratio to GAPDH. Data are expressed as means ± SD (n = 4–6). ^#^P < 0.05 versus the injury group. *P < 0.05 versus the previous group.

The effects of AC-YVAD-CMK treatment on the NLRP3 inflammasome were evaluated using western blotting and real-time polymerase chain reaction (RT-PCR). Expression of the NLRP3 protein was elevated markedly by acute gastric injury (P < 0.01). Pretreatment with AC-YVAD-CMK had no effect on this increase compared with the control group. Caspase-1 activation (i.e. the formation of cleaved caspase-1) was inhibited significantly in a dose-dependent manner by pretreatment with AC-YVAD-CMK (1.25, 6.25, and 12.5 μmol/kg, P < 0.05, Fig. 1A). The results of the RT-PCR analysis showed that there was no inhibitory effect of AC-YVAD-CMK pretreatment on the expression of ASC mRNA compared with the injury-alone group (P > 0.05, Fig. 1B).

### Decreased caspase-1 activity, but not acid secretion, protected against cold-restraint stress-induced gastric ulcer

**Figure 2:**
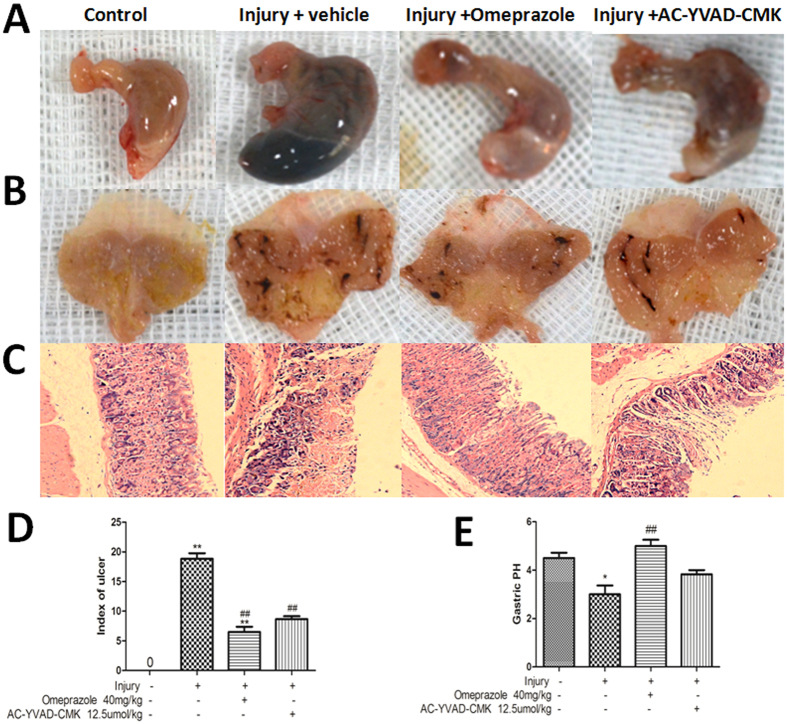
Decreased caspase-1, but not acid secretion, protects against cold-restraint stress-induced gastric injury. Photographs of gastric mucosa were taken 8 h after cold-restraint stress injury. Pretreatment with (**A**) omeprazole or (**B**) AC-YVAD-CMK diminished the number of haemorrhagic erosions. (**C**) Gastric mucosa histopathological changes are shown following H&E staining (magnification, 100×). (**D,E**) The gastric ulcer index and mucosal pH were measured to confirm the protective effect of omeprazole and AC-YVAD-CMK on gastric ulcer formation and acid secretion following cold-restraint stress injury. Data are expressed as means ± SD (n = 6). ^##^P < 0.01 versus the injury-alone group. **P < 0.01, *P < 0.05 versus the previous group.

Histopathological evidence showed that cold-restraint stress induced multiple haemorrhagic gastric erosions and ulcers that were reduced significantly by pretreatment with either omeprazole or AC-YVAD-CMK (Fig. 2A–C). Quantitatively, the gastric ulcer index was reduced from 18.83 ± 2.32 in the injury-alone group to 8.67 ± 1.21 and 6.50 ± 2.17 in the injury + AC-YVAD-CMK and injury + omeprazole groups, respectively (P < 0.01). There was no significant difference between the AC-YVAD-CMK and omeprazole groups (P > 0.05, Fig. 2D).

Gastric pH was measured to study the effect of inhibiting caspase-1 or gastric acid secretion on cold-restraint stress-induced gastric injury. Gastric mucosal pH was 1.33 ± 0.52 in the injury-alone group, and 3.50 ± 0.55 and 2.00 ± 0.63 in mice pretreated with omeprazole or AC-YVAD-CMK, respectively (Fig. 2). This indicated that gastric acid production was effectively inhibited by omeprazole, but not by AC-YVAD-CMK (P > 0.05, Fig. 2E), and suggested that changes in gastric pH were not responsible for the observed protection by AC-YVAD-CMK.

### Pretreatment with glybenclamide or AC-YVAD-CMK protected against acute gastric ulcer by diminishing NLRP3 inflammasome activities

**Figure 3:**
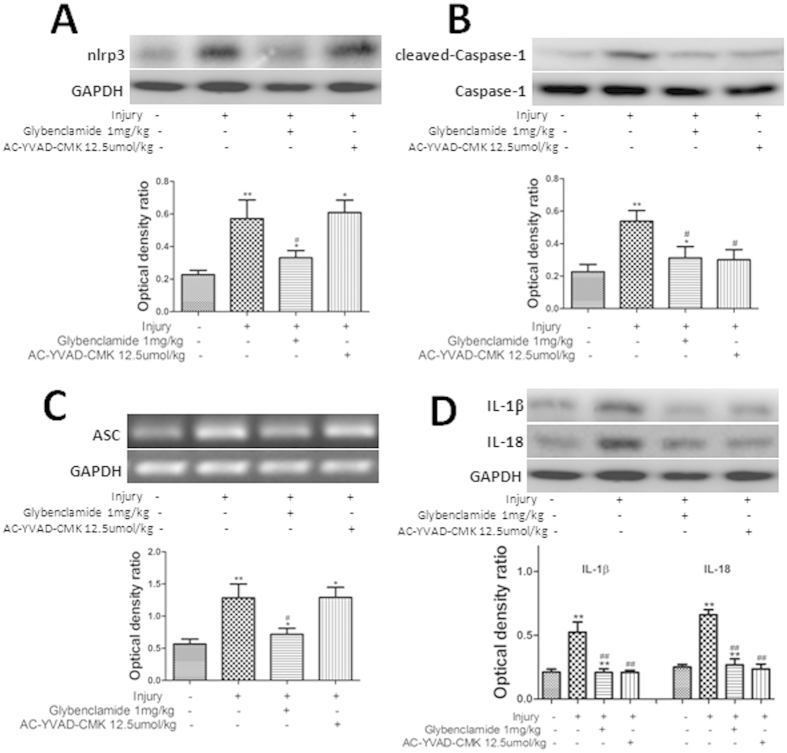
Pretreatment with glybenclamide or AC-YVAD-CMK diminished NLRP3 inflammasome activities. (**A,B**) The expression of NLRP3 and cleaved-caspase-1 proteins was assessed by western blotting. (**C**) The expression of ASC mRNA following pretreatment with AC-YVAD-CMK or glybenclamide was determined using RT-PCR. (**D,E**) Levels of IL-1β and IL-18 were determined by western blotting. Data are expressed means ± SD (n = 4). *P < 0.05, ^##^P < 0.01 versus the injury group. **P < 0.01, *P < 0.05 versus the previous group.

Western blots showed that the expression of NLRP3 and cleaved caspase-1 proteins was elevated markedly by acute gastric injury (Fig. 3A). Pretreatment with glybenclamide, but not AC-YVAD-CMK, significantly reduced the high levels of NLRP3 protein. Caspase-1 activation was inhibited significantly by pretreatment with either AC-YVAD-CMK or glybenclamide (P < 0.05, Fig. 3B). RT-PCR showed that there was no effect of AC-YVAD-CMK pretreatment on the expression of ASC mRNA compared with the injury-alone group. However, pretreatment with glybenclamide reduced the high levels of ASC mRNA expression caused by the injury (P < 0.05 Fig. 3C). Western blot analyses showed that gastric levels of IL-1β and IL-18 were elevated significantly after cold-restraint stress injury. Both AC-YVAD-CMK and glybenclamide reduced the elevated levels of IL-1β and IL-18, significantly (P < 0.01 Fig. 3D).

### The NLRP3 inflammasome negatively influenced the inflammatory response following cold-restraint stress-induced gastric injury

**Figure 4:**
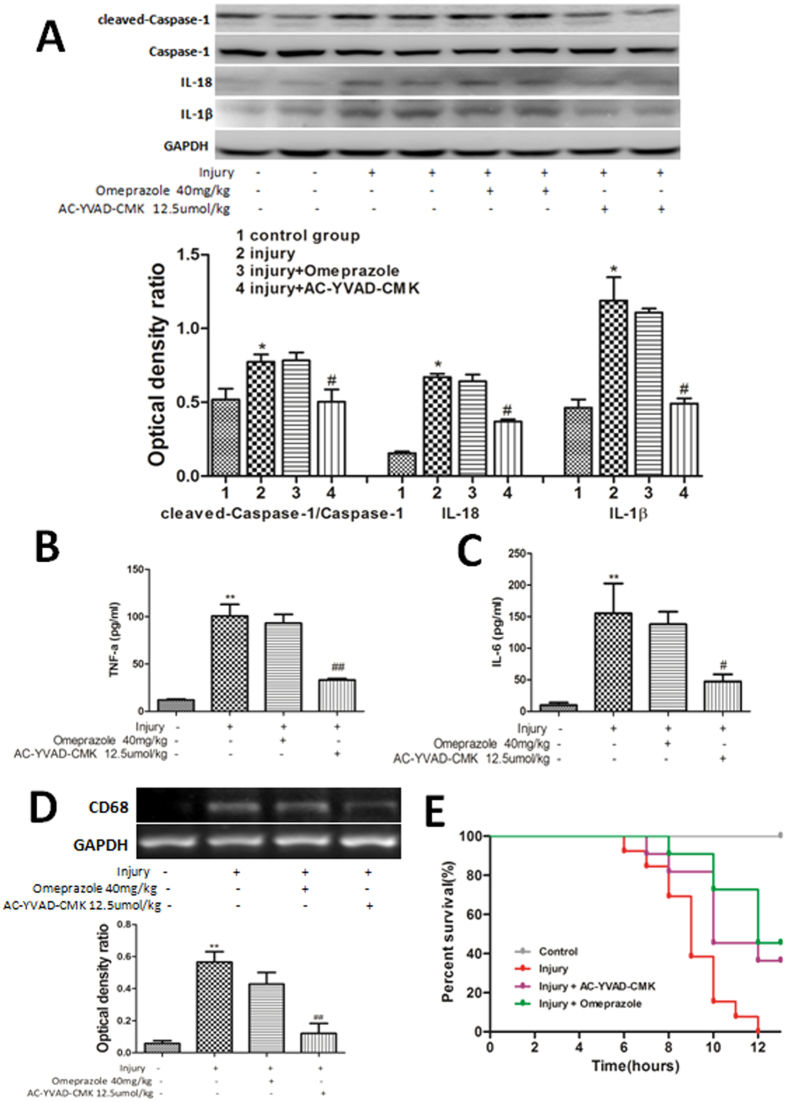
Inhibiting caspase-1 activity alleviated acute inflammatory responses and improved survival following cold-restraint stress injury. (**A**) The expression of cleaved caspase-1, IL-18, and IL-1β proteins was measured by western blotting. Protein levels were quantified by densitometry and are expressed as the optical density ratio to GAPDH (n = 6). ELISA results showed that mice pretreated with AC-YVAD-CMK had lower levels of TNF-α (**B**) and IL-6 (**C**) compared with those in the injury-alone group (n = 6). (**D**) CD68 mRNA levels in the gastric mucosa were measured by RT-PCR (n = 4). The mean survival times (range) of mice in the injury-alone, injury + omeprazole, and injury + AC-YVAD-CMK groups were 9.08 (8.20–9.95), 11.73 (10.78–12.67), and 10.82 (9.61–12.02), respectively. (**E**) The survival analysis showed that mice pretreated with omeprazole (P < 0.01) or AC-YVAD-CMK (P < 0.05) had lower mortality rates than mice receiving vehicle (n = 11 in each group). Data are expressed as means ± SD. ^##^P < 0.01, ^#^P < 0.05 versus the injury-alone group. **P < 0.01, *P < 0.05 versus the previous group.

Western blot analysis showed that the formation of cleaved caspase-1 protein was elevated markedly by acute gastric injury. Pretreatment with AC-YVAD-CMK, but not omeprazole, significantly inhibited this cleavage (P < 0.05 Fig. 4A). Both IL-1β and IL-18 increased after cold-restraint stress-induced injury. These increases were reduced significantly by pretreatment with AC-YVAD-CMK (P < 0.05), but not omeprazole (P > 0.05), compared to the values reported for the injury-alone group (Fig. 4A). Enzyme-linked immunosorbent assay (ELISA) results showed that mice pretreated with AC-YVAD-CMK, but not omeprazole, had lower levels of TNF-α (P < 0.01) and IL-6 (P < 0.05) in serum than did the injury-alone group (Fig. 4B,C).

To assess the infiltration of monocytes/macrophages, the expression of CD68 mRNA was measured in gastric tissues^15^. The high levels of CD68 mRNA expression induced by cold stress injury were reduced significantly by pretreatment with AC-YVAD-CMK (P < 0.01) but not omeprazole (Fig. 4D).

Kaplan–Meier survival curves showed that treatment of mice with omeprazole (P < 0.01) or AC-YVAD-CMK (P < 0.05) 30 min before cold-stress injury decreased the mortality rate compared with mice that received vehicle (Fig. 4E). The mean survival times (range) of mice in the injury, injury + omeprazole, and injury + AC-YVAD-CMK groups were 9.08 (8.20–9.95), 11.73 (10.78–12.67), and 10.82 (9.61–12.02) hours, respectively.

### Inhibiting caspase-1 activity in the NLRP3 inflammasome alleviated cold-restraint stress-induced gastric cell apoptosis

**Figure 5:**
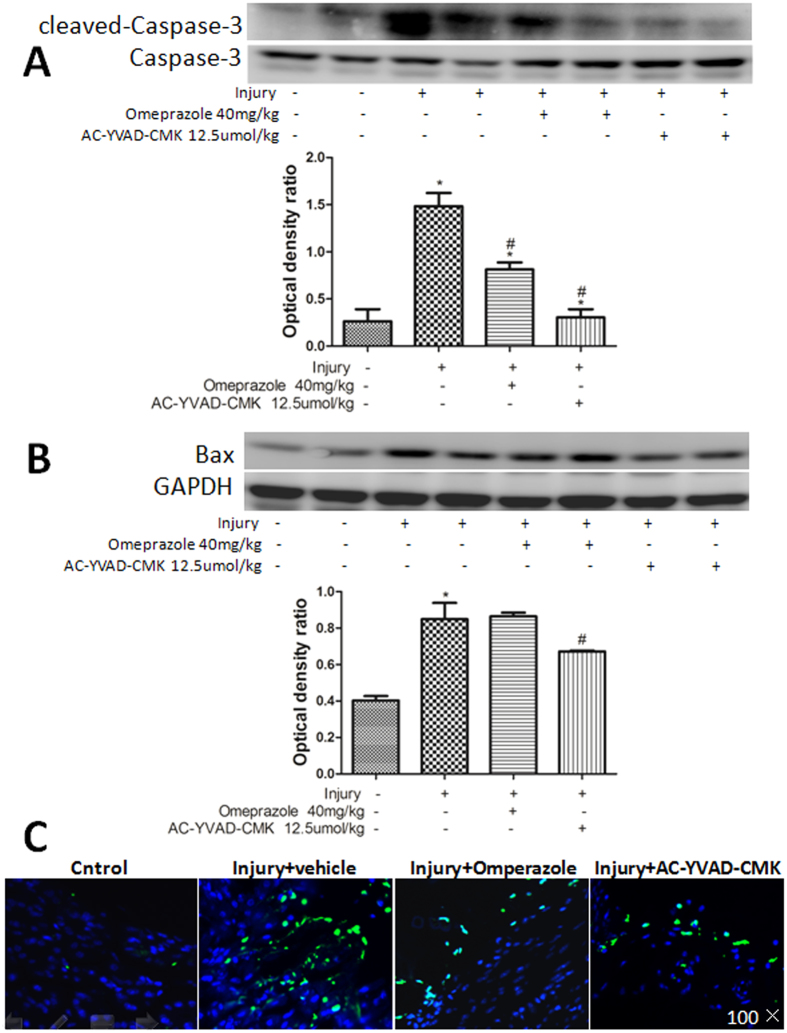
Inhibiting caspase-1 reduced cold-restraint stress-induced gastric cell apoptosis. The expression of cleaved caspase-3 and caspase-3 (**A**), and Bax (**B**) proteins was measured by western blotting. Protein levels were quantified by densitometry and are expressed as the optical density ratio to GAPDH. (**C**) Apoptotic cells were observed in the nucleus of the gastric mucosa by TUNEL staining (magnification, 100×). Data are expressed as means ± SD (n = 4). ^#^P < 0.05 versus the injury-alone group. *P < 0.05 versus the previous group.

In the mouse cold-restraint stress model, apoptosis of gastric cells is a common pathological characteristic^16^,^17^. Protein levels of cleaved caspase-3, caspase-3, and Bax were measured by western blotting, and cell apoptosis by terminal deoxynucleotidyl transferase-mediated dUTP nick end labeling (TUNEL) staining. Pretreatment with AC-YVAD-CMK reduced the high levels of active caspase-3 in the injury group (P < 0.05, Fig. 5A). The expression of Bax was high in both the injury-alone and injury + omeprazole groups. The expression of Bax was reduced slightly, but significantly, by pretreatment with AC-YVAD-CMK (P < 0.05 Fig. 5B). The TUNEL results revealed a large number of apoptotic cells located on top of the mucosal epidermis in the injury-alone group. Pretreatment with AC-YVAD-CMK or omeprazole reduced the number of apoptotic cells (Fig. 5C).

### Effects of AC-YVAD-CMK on the P38 MAPK/NF-κB signalling pathways in cold-restraint stress injury

**Figure 6:**
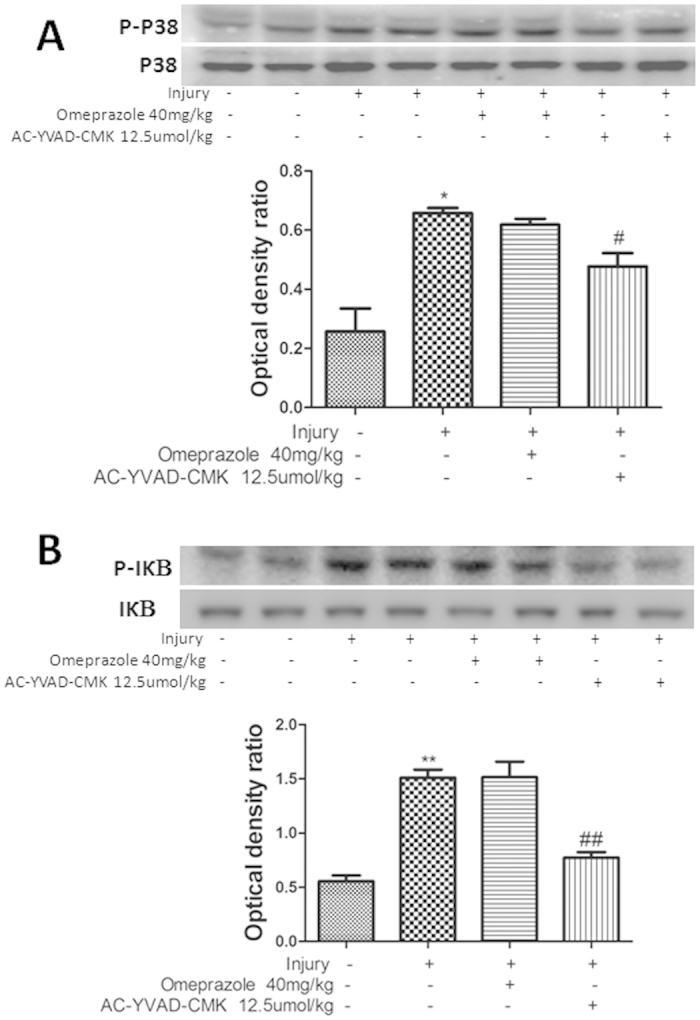
Effects of AC-YVAD-CMK on P38/NF-κB signalling pathways. The expression of P-P38 and P38 (**A**), and P-IκB and IκB (**B**) proteins was measured by western blotting. Protein levels were quantified by densitometry and are expressed as the optical density ratio to GAPDH. Data are presented as means ± SD (n = 4). ^##^P < 0.01, ^#^P < 0.05 versus the injury-alone group. **P < 0.01, *P < 0.05 versus the previous group.

The MAPK/NF-κB signalling pathways play an important role in acute gastric injury in mice^1^. Pretreatment with AC-YVAD-CMK decreased the expression of phospho (P)-P38 protein compared to the injury-alone and injury + omeprazole groups (P < 0.05 Fig. 6A). Furthermore, pretreatment with AC-YVAD-CMK alleviated the overexpression of P-IκB induced by cold-restraint stress, compared with the injury-alone and injury + omeprazole groups (P < 0.01 Fig. 6B).

### The protective effect of omeprazole and AC-YVAD-CMK against ethanol-induced gastric injury

**Figure 7:**
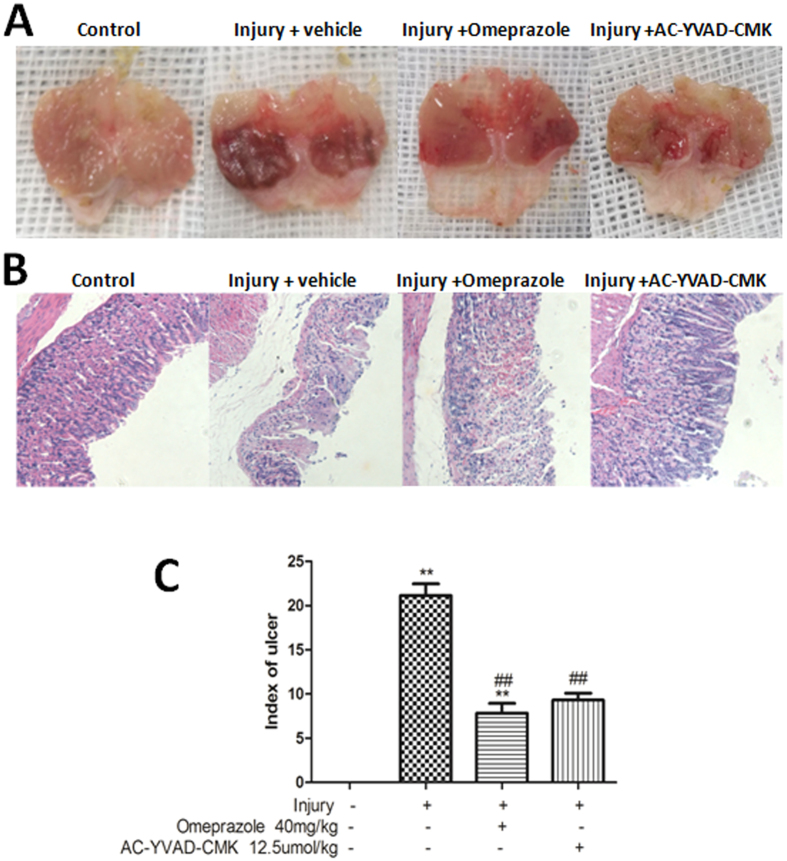
The protective effects of inhibiting caspase-1 against ethanol-induced gastric injury. Photographs of gastric mucosa were taken 4 h after gastric injury was induced by ethanol. (**A**) Pretreatment with omeprazole or AC-YVAD-CMK prevented the formation of multiple haemorrhagic erosions. (**B**) Histopathological changes to the gastric mucosa were evaluated following H&E staining (magnification, 100×). (**C**) The gastric ulcer index was measured to quantify the protective effects of omeprazole and AC-YVAD-CMK. Data are presented as means ± SD (n = 6). ^##^P < 0.01, ^#^P < 0.05 versus the injury-alone group. **P < 0.01, *P < 0.05 versus the previous group.

Light micrographs and haematoxylin and eosin (H&E) staining showed that ethanol induced multiple haemorrhagic erosions and ulcers that were alleviated significantly by pretreatment with either omeprazole or AC-YVAD-CMK (Fig. 7A,B). The gastric ulcer index was reduced from 21.2 ± 3.2 in the injury-alone group to 9.3 ± 1.9 and 7.83 ± 2.71 in the injury + AC-YVAD-CMK and injury + omeprazole groups, respectively (P < 0.01). There was no significant difference between the groups of mice treated with AC-YVAD-CMK and omeprazole (P > 0.05, Fig. 7C).

## Discussion

Gastric ulcers are a common complication of patients in critical condition and available treatments remain inadequate. A hyper-inflammatory response with an increased production of cytokines is an important characteristic of acute gastric injury^18^,^19^. Inflammasomes are important components of the innate immune system, and regulate the inflammatory processes and cell death in response to a diverse array of stimuli. Regulation of inflammasomes can affect the balance between the production of anti- and pro-inflammatory cytokines^20^. Higashimori *et al*. demonstrated that the NLRP3 inflammasome regulated the damage, as well as caspase-1 activation and IL-1β processing, that played a crucial role in the enteropathy induced by non-steroidal anti-inflammatory agents^21^. However, there is little information about the effect of inhibiting caspase-1 in the NLRP3 inflammasome on acute gastric injury. In the current study, we inhibited caspase-1 in NLRP3 inflammasomes and evaluated the effect on experimental acute gastric injury in mice.

The major etiological factors of acute gastric ulcer include gastric acid, stress, alcohol consumption, abnormal motility, and *H. pylori* infection^18^,^22^. Omeprazole treatment ensures a continuous suppression of gastric acid secretion, and its therapeutic benefit on gastric ulcer is well-established^23^. In the present study, omeprazole was used as a counterpoint for a treatment to inhibit caspase-1. We found that pretreatment with either AC-YVAD-CMK or omeprazole mitigated cold-restraint stress- and ethanol-induced gastric mucosal histopathological changes. However, pretreatment with AC-YVAD-CMK protected mice from developing acute gastric ulcers and multiple haemorrhagic lesions without raising the gastric mucosal pH.

An acute inflammatory response with over-production of pro-inflammatory cytokines is associated with the severity of acute gastric ulcer, and might augment further injury^4^,^5^,^6^. In this study, we observed an increase of TNF-α, IL-1β, IL-6, and IL-18 following cold-restraint stress injury. These changes were reduced by treatment with a caspase-1 inhibitor. Furthermore, we found that there was an increased expression of CD68 mRNA in the gastric mucosa after cold-restraint stress injury. These findings confirmed that there was an inflammatory response following acute gastric injury. The hyper-inflammation was alleviated by AC-YVAD-CMK pretreatment, suggesting that preventing caspase-1 activation has an anti-inflammatory effect on acute gastric injury.

Apoptosis is another major characteristic of acute gastric injury^24^. Tsukimi *et al*. reported that high levels of pro-inflammatory cytokines, particularly TNF-α, induced cell apoptosis by activating the caspase family of proteases^25^. Therefore, we evaluated the effects of AC-YVAD-CMK pretreatment on the levels of Bax, cleaved caspase-3, and caspase-3, components of the mitochondrial apoptotic pathway. Our results demonstrated that mice pretreated with AC-YVAD-CMK had a lower expression of Bax and cleaved caspase-3 proteins. Furthermore, we found that the increased number of apoptotic cells located on the epidermis of the gastric mucosal membrane following cold-restraint stress injury was reduced by pretreatment with AC-YVAD-CMK.

**Figure 8:**
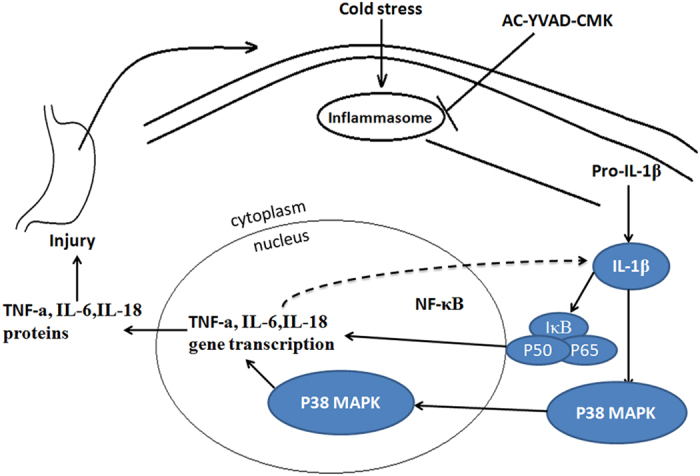
Proposed mechanism underlying the protective effects of caspase-1 inhibition on cold-constraint stress-induced gastric injury. The NLRP3 inflammasome recognizes danger signals of cold stress and becomes activated. AC-YVAD-CMK pretreatment prevents activation of the inflammasome, inhibiting the maturation of IL-1β and lessening the production of other pro-inflammatory cytokines such as TNF-α, IL-6, and IL-18, which are associated with P38 and NF-κB signalling pathways.

NF-κB, an important transcription factor, is ubiquitous in the cytoplasm as a complex with its inhibitory protein, IκB. After activation by various stimuli, IκB dissociates from NF-κB and undergoes ubiquitination. NF-κB is then translocated to the nucleus and activates signalling pathways. Many previous studies demonstrated that NF-κB plays an important role in regulating inflammatory processes. P38 MAPK is another important signalling pathway that participates in intracellular signal transduction and the production of chemokines and pro-inflammatory cytokines^26^. Li *et al*. reported that anti-inflammatory effects might be associated with the downregulation of NF-κB activity in ethanol-induced acute gastric ulcer in mice^1^. In the present study, P-P38 and P-IκB expression were decreased following cold-restraint stress injury by pretreatment with AC-YVAD-CMK, but not omeprazole. Furthermore, AC-YVAD-CMK pretreatment inhibited the production of IL-1β, an early pro-inflammatory cytokine that stimulates the production of other cytokines such as TNF-α and IL-6^27^. Importantly, we found that inhibiting caspase-1 activity decreased P38 MAPK and NF-κB signalling activities, and might be associated with the inhibition of IL-1β production (Fig. 8).

The present study has two main limitations. First, although the NLRP3 inflammasome inhibitor glybenclamide was used as a control, all experiments involved pharmacologic agents that have inherent specificity limitations. Future studies with NLRP3 and caspase-1 knockout mice would provide more direct information on the roles of these factors in gastric injury. Second, because there are no vitro models for studying acute gastric injury, we could not investigate the mechanism of inhibiting caspase-1 on acute gastric injury using an isolated cell model or cell lines.

## Materials and Methods

### Animals

Male C57BL/6 mice weighing 21–25 g were purchased from the Experimental Animal Centre, Second Military Medical University (SMMU), Shanghai, China. Mice were housed under controlled conditions (23 ± 2 °C and 50 ± 5% humidity) and fed standard laboratory food. All animal experiments were approved by the Institutional Animal Care and Use Committee of the SMMU in strict accordance with the guidelines for the Care and Use of Laboratory Animals published by the U.S. National Institutes of Health (publication No. 96–01).

### Reagents

AC-YVAD-CMK was obtained from Cayman Chemical Company (Ann Arbor, MI, USA). Glybenclamide was purchased from Novus Biologicals (Littleton, CO, USA). Omeprazole was provided by the SMMU affiliated Changhai Hospital (Shanghai, China). ELISA kits for IL-6 and TNF-α were purchased from eBioscience Systems (San Diego, CA, USA). Rabbit monoclonal antibodies against caspase-3, cleaved caspase-3, Bax, P38, P-P38, P-IκB, IκB, and GAPDH were obtained from Cell Signaling Technology (Beverly, MA, USA). Rabbit monoclonal antibodies against IL-18 and IL-1β were obtained from Bioworld Biotechnology (Wuhan, Hubei, China). Goat monoclonal antibodies against cleaved caspase-1 and caspase-1, and horseradish peroxidase-conjugated rabbit anti-goat and goat anti-rabbit antibodies, were obtained from Santa Cruz Biotechnology (Dallas, TX, USA). TRIzol reagent was obtained from Invitrogen (Carlsbad, CA, USA). pH diagnostic test strips were purchased from Xinxing Biotechnology (Hangzhou, Zhejiang, China).

### Models of mouse acute gastric injury

**Figure 9:**
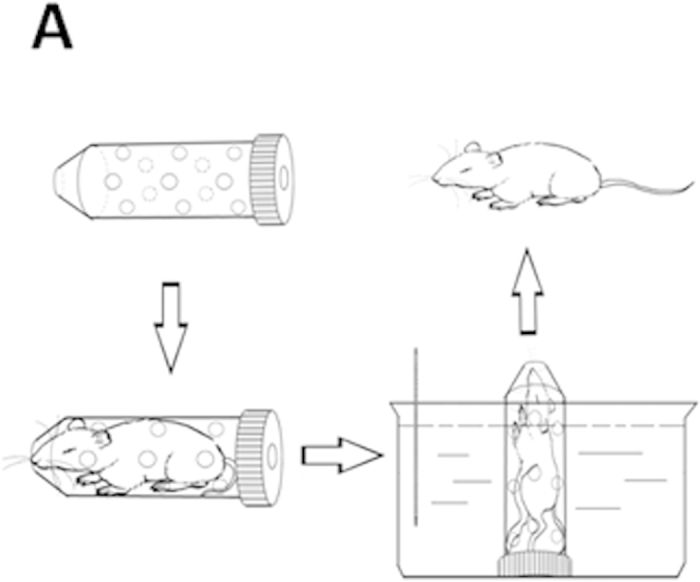
The cold-restraint stress-induced acute gastric injury model in mice. (**A**) A simple plastic device was designed by our department and has received a Chinese patent.

To generate cold-restraint stress-induced acute gastric injury in mice, a plastic device was designed by our department (Chinese Patent ZL 201420111867.X, Fig. 9A). After fasting for 24 h, mice were restrained in the device and immersed to the depth of the xiphoid process in water at 20 °C for 8 h^28^. Gastric injury was also induced with ethanol to further study the protective effects of inhibiting caspase-1 in the NLRP3 inflammasome. After fasting for 24 h, 100% ethanol (0.1 mL/kg) was administered to mice intragastrically. Mice in the control group received phosphate-buffered saline (PBS) instead of ethanol^29^. Animals were sacrificed 4 h after treatment, and gastric tissues and blood samples were collected.

### Experimental protocols

Twenty-four mice were used to induce acute gastric injury with cold-restraint stress. These mice were divided randomly into four groups of six. (1) Control: each animal was injected intraperitoneally (i.p.) with dimethyl sulphoxide (DMSO) and PBS (1:9, v/v, 10 mL/kg). (2) Injury alone: DMSO and PBS (1:9, v/v, 10 mL/kg) were injected i.p. 30 min before cold-restraint. (3) Injury + AC-YVAD-CMK: AC-YVAD-CMK was freshly dissolved in DMSO and PBS (1:9, v/v), and administered i.p. (12.5 μmol/kg) 30 min before cold-restraint. (4) Injury + omeprazole: Omeprazole was dissolved in PBS and injected i.p. (40 mg/kg) 30 min before cold-restraint.

The inhibitory effect of AC-YVAD-CMK on caspase-1 activity in the NLRP3 inflammasome was assessed by treating mice in the injury group with different concentrations (1.2, 6.25, or 12.5 μmol/kg) dissolved in DMSO and PBS (1:9, v/v). To determine the effect of silencing caspase-1 in the NLRP3 inflammasome via a different mechanism, glybenclamide (that blocks the maturation of caspase-1 and pro-IL-1β) was dissolved in DMSO and PBS (1:9, v/v), and given i.p. (1 mg/kg) 30 min before cold-restraint stress-induced gastric injury.

Ethanol-induced acute gastric injury was induced in twenty-four mice divided randomly into four equal groups and treated as described for cold-restraint stress. In both experiments, animals were sacrificed by a single i.p dose of pentobarbital sodium (300 mg/kg). Blood samples were collected from the left ventricle. After taking photographs of the gastric tissue and measuring the gastric pH, gastric tissue from each mouse was split with one moiety being used for histological analysis and the other stored at −80 °C for biochemical analyses.

### H&E staining

For histological analysis, a portion of the mouse gastric tissue was fixed in 10% neutral-buffered formalin. Five-micrometre-thick sections were embedded into paraffin, sliced, and stained with H&E. Histopathological examinations were carried out using a light photomicroscope.

### Pro-inflammatory cytokine assays

The acute phase of inflammation after mouse cold-restraint stress-induced gastric injury was assessed by measuring the serum pro-inflammatory cytokines, IL-6 and TNF-α, using commercial ELISA kits according to the manufacturer’s instructions. Results were determined at 450 nm using a microplate reader (Corning Corporation, Corning, NY, USA).

### TUNEL assay

Apoptosis of gastric mucosal cells was measured with a commercial TUNEL assay kit (Roche Diagnostics, Indianapolis, IN, USA). The assays were carried out according to the manufacturer’s instructions. TUNEL-positive cells were identified and counted using a fluorescence microscope^30^.

### RNA extraction and RT-PCR measurements

RT-PCR was performed, as reported previously, to measure the expression of ASC mRNA in the NLRP3 inflammasome and CD68 positive gastric cells^31^,^32^. Total RNA was extracted from gastric tissues using TRIzol reagent (Life Technologies, Carlsbad, CA, USA) and reverse transcribed using a 50 μL oligo (dT) system. After heating at 95 °C for 4 min, cDNA amplification was performed in a thermal cycler using the following conditions: 95 °C for 30 s, 56 °C for 30 s, and 73 °C for 30 s for 29 cycles (Biometra, Göttingen, Germany). The products were separated using gel electrophoresis and analysed with a scanning densitometer (Alpha Innotech, Santa Clara, CA, USA).

Primers for RT-PCR were ASC forward, 5′-AGACATGGGCTTACAGGA-3′, reverse, 5′-CTCCCTCATCTTGTCTTGG-3′. CD68 forward, 5′-CCCACAACTGTCACTCATAACC-3′, reverse, 5′-TCTGAAATCACAAGAATGCTCC-3′. GAPDH forward, 5′-AGAACATCATCCCTGCATCC-3′, reverse, 5′-TCCACCACCCTGTTGCTGTA-3′.

### Western blot analyses

About 300 mg of gastric tissue was homogenized in 1.2 mL tissue protein extraction reagent. Homogenates were centrifuged at 10,000 rpm for 20 min at 4 °C. The protein concentrations of the supernatants were determined with the bicinchoninic acid assay. Western blot analyses were carried out for caspase-3, cleaved caspase-3, Bax, caspase-1, cleaved caspase-1, P38, P-P38, P-IκB, IκB, IL-18, IL-1β, and GAPDH as described previously^33^.

### Statistical analyses

Statistical calculations were carried out using SPSS 16.0 (IBM, Chicago, IL, USA). Results are expressed as means ± standard deviation (SD). Multiple comparisons among groups were analysed using either nonparametric tests or one-way analysis of variance. Survival was plotted using the Kaplan-Meier method. Differences among groups were analysed using the log-rank test. Differences with P < 0.05 were considered statistically significant.

## Conclusions

In summary, the caspase-1 specific inhibitor, AC-YVAD-CMK, protected against experimental acute gastric injury in mice. This protective effect might involve silencing NLRP3 inflammasome activities, and attenuating inflammatory processes and apoptosis. This would be similar to the underlying mechanism associated with NF-κB and P38 MAPK signalling pathways.

## Additional Information

**How to cite this article**: Zhang, F. *et al*. The caspase-1 inhibitor AC-YVAD-CMK attenuates acute gastric injury in mice: involvement of silencing NLRP3 inflammasome activities. *Sci. Rep.* **6**, 24166; doi: 10.1038/srep24166 (2016).
